# Estimation of ambient PM_2.5_ in Iraq and Kuwait from 2001 to 2018 using machine learning and remote sensing

**DOI:** 10.1016/j.envint.2021.106445

**Published:** 2021-02-19

**Authors:** Jing Li, Eric Garshick, Jaime E. Hart, Longxiang Li, Liuhua Shi, Ali Al-Hemoud, Shaodan Huang, Petros Koutrakis

**Affiliations:** a Department of Environmental Health, Harvard T.H. Chan School of Public Health, Boston 02115, USA; b Pulmonary, Allergy, Sleep, and Critical Care Medicine Section, Medical Service, VA Boston Healthcare System, Boston, MA 02132, USA; c Channing Division of Network Medicine, Department of Medicine, Brigham and Women’s Hospital and Harvard Medical School, Boston, MA 02115, USA; d Department of Environmental Health, Rollins School of Public Health, Emory University, Atlanta, GA 30322, USA; e Crisis Decision Support Program, Environment and Life Sciences Research Center, Kuwait Institute for Scientific Research, Safat 13109, Kuwait

**Keywords:** PM_2.5_, Aerosol optical depth (AOD), Visibility, High resolution, Exposure

## Abstract

Iraq and Kuwait are in a region of the world known to be impacted by high levels of fine particulate matter (PM_2.5_) attributable to sources that include desert dust and ambient pollution, but historically have had limited pollution monitoring networks. The inability to assess PM_2.5_ concentrations have limited the assessment of the health impact of these exposures, both in the native populations and previously deployed military personnel. As part of a Department of Veterans Affairs Cooperative Studies Program health study of land-based U.S. military personnel who were previously deployed to these countries, we developed a novel approach to estimate spatially and temporarily resolved daily PM_2.5_ exposures 2001–2018. Since visibility is proportional to ground-level particulate matter concentrations, we were able to take advantage of extensive airport visibility data that became available as a result of regional military operations over this time period. First, we combined a random forest machine learning and a generalized additive mixed model to estimate daily high resolution (1 km × 1 km) visibility over the region using satellite-based aerosol optical depth (AOD) and airport visibility data. The spatially and temporarily resolved visibility data were then used to estimate PM_2.5_ concentrations from 2001 to 2018 by converting visibility to PM_2.5_ using empirical relationships derived from available regional PM_2.5_ monitoring stations. We adjusted for spatially resolved meteorological parameters, land use variables, including the Normalized Difference Vegetation Index, and satellite-derived estimates of surface dust as a measure of sandstorm activity. Cross validation indicated good model predictive ability (R^2^ = 0.71), and there were considerable spatial and temporal differences in PM_2.5_ across the region. Annual average PM_2.5_ predictions for Iraq and Kuwait were 37 and 41 μg/m^3^, respectively, which are greater than current U.S. and WHO standards. PM_2.5_ concentrations in many U.S. bases and large cities (e.g. Bagdad, Balad, Kuwait city, Karbala, Najaf, and Diwaniya) had annual average PM_2.5_ concentrations above 45 μg/m^3^ with weekly averages as high as 150 μg/m^3^ depending on calendar year. The highest annual PM_2.5_ concentration for both Kuwait and Iraq were observed in 2008, followed by 2009, which was associated with extreme drought in these years. The lowest PM_2.5_ values were observed in 2014. On average, July had the highest concentrations, and November had the lowest values, consistent with seasonal patterns of air pollution in this region. This is the first study that estimates long-term PM_2.5_ exposures in Iraq and Kuwait at a high resolution based on measurements data that will allow the study of health effects and contribute to the development of regional environmental policies. The novel approach demonstrated may be used in other parts of the world with limited monitoring networks.

## 1. Introduction

Since 2001, many U.S. military personnel and coalition military personnel have been deployed in support of operations in the Southwest Asia and experienced with frequent exposure to high concentrations of fine particulate matter (PM_2.5_) attributable to large arid and semiarid areas, local pollution from vehicles and industry, oil exploration and refining, and refuse-burning, including burn pits (Alolayan et al., 2013; Engelbrecht et al., 2009; Garshick et al., 2019). Epidemiologic studies based on military health encounter data have demonstrated more frequent post-deployment health encounters than non-deployed personnel for respiratory symptoms and for airway disease, predominantly asthma (Abraham et al., 2014; Pugh et al., 2016; Abraham et al., 2012; Sharkey et al., 2016). There is concern that deployers may have experienced reductions in pulmonary function or other health effects attributable to exposure (U.S. Institute of Medicine, 2011; Garshick et al., 2019). Moreover, exposure to air pollution has been directly linked to health effects worldwide (Pappin and Hakami, 2013; Stafoggia et al., 2017; Brauer et al., 2016; Wang et al., 2020; Zhang et al., 2021). Unlike health studies assessing PM-related health effects in the US, Europe, and parts of Asia, the lack of a comprehensive network of regional monitoring stations in countries such as Iraq and Kuwait has hindered the design of health effect studies, both in the native populations and previously deployed military personnel.

Visibility data are routinely collected at airports or meteorology stations throughout the world. The relationship between visibility and PM_2.5_ is relatively stable due to the light extinction (scattering and absorption) effects of particles with sizes similar to the wavelengths of visible light, particularly in arid areas (Abbey et al., 1995; Zhao et al., 2017; Liu et al., 2017; Vajanapoom et al., 2001). Visibility has been used previously to estimate historical ground-level PM_2.5_ exposures in China prior to the establishment of a PM_2.5_ monitoring network (Liu et al., 2017). Masri et al. (2017) conducted a pilot study that demonstrated the feasibility of using airport visibility data in Southwest Asia and Afghanistan. Visibility-based approach takes advantage of the large database of historical visibility collected by airport or meteorology stations, but the spatial resolution of estimated PM_2.5_ is still coarse for epidemiological studies.

Satellite-based aerosol optical depth (AOD), a measure of light attenuation in the column of air from Earth’s surface to the top of atmosphere which is affected by meteorology and land conditions (Paciorek and Liu, 2009), has been widely used to estimate spatiotemporally resolved PM_2.5_ exposure, given its extensive spatial coverage, high spatial resolution, and reliable repeated daily observations (Liu et al., 2005; Paciorek et al., 2008; Chudnovsky et al., 2012; Kloog et al., 2014; Beloconi et al., 2016; Brokamp et al., 2017; Huang et al., 2018; Di et al., 2019). The satellite-based approach has been applied to different study areas, such as the United States (Kloog et al., 2014; Di et al., 2016), China (Xue et al., 2019), and Europe (Beloconi et al., 2018).

Quantifying a relationship between AOD and PM_2.5_ usually involves incorporating ground-level PM_2.5_ measurements, metrological variables, and geographic covariates at high spatial and temporal resolutions. However, this method has not been applied to areas with a paucity of PM_2.5_ monitoring sites due to the lack of spatial coverage. There are no monitoring networks in the Middle East designed to provide comprehensive measurements. Instead of using ground-level PM_2.5_ measurements, some studies use a Chemical Transport Model (CTM) or observational meteorological values to simulate the relationship between AOD and PM_2.5_ globally (van Donkelaar et al., 2015; Lin et al., 2018). However, CTMs rely on accurate emission inventories, which are poorly developed in the Middle East. Therefore, current models that estimate PM_2.5_ globally using CTM inventories are unreliable in this region. Until our efforts, there have been no previous studies assessing historical PM_2.5_ exposures at high spatial resolution in the Middle East based on measurement data.

As part of a Department of Veterans Affairs Cooperative Studies Program health study of land-based U.S. military personnel, we predicted spatially and temporally resolved PM_2.5_ concentrations by a multi-staged approach. First, we used a hybrid machine learning model and incorporated multiple variables (including satellite-based AOD data, land-use terms, and meteorological variables) to predict high spatiotemporal resolution visibility, as visibility data throughout the region were available as result of military airport activity. We then derived relationships between daily PM_2.5_ and visibility using existing monitoring stations in the region to convert visibility to PM_2.5_. In the absence of extensive air quality monitoring sites, this approach leverages historical visibility data, satellite AOD, and existing regional PM_2.5_ monitoring stations to understand the intensity and spatiotemporal variability in PM_2.5_ exposures over Iraq and Kuwait.

## 2. Material and methods

### 2.1. Data collection

#### 2.1.1. AOD data

We collected 1 km × 1 km AOD data for Iraq and Kuwait between January 1st, 2001 through December 31st, 2018 from the MODIS instrument, which is onboard Terra and Aqua, two Earth Observing System (EOS) satellites (Salomonson et al., 1989). Terra was launched in December 1999, and Aqua was launched in May 2002. The local crossing time over Kuwait and Iraq for Terra is approximately 10:30, while for Aqua it is approximately 13:30. We used Collection-6 Multi-Angle Implementation of Atmospheric Correction (MAIAC) AOD data (MCD19A2), which includes a correction that adjusts for reflection of bright light from desert regions, from the Terra for 2001–2018 and the Aqua for 2003–2018. (Lyapustin et al., 2018) Four sections (tiles) of the NASA global AOD database (tiles h22v05, h22v06, h21v05, h21v06) that included approximately 360,000 grid cells for each tile were used. We extracted AOD at 550 nm that met MODIS quality assurance criteria (flag of “good”), and data with extreme values (above 4) were excluded (Di et al., 2016). Due to cloud cover and technical factors such as imaging over some bright surfaces and cloud, AOD has missing approximately 40% (see Fig. S2 in supplement) (Xiao et al., 2017). Therefore, we calculated linear associations between the Aqua and Terra AOD for each day and imputed the missing MAIAC AOD values when values from only one instrument was available (Huang et al., 2018). Grid cells over large water bodies were excluded (https://www.diva-gis.org/datadown) (Fig. S1). The average AOD from Aqua and Terra was used for modeling.

#### 2.1.2. Visibility data

Hourly visibility data for Kuwait and Iraq were provided by the U.S. Air Force 14th Weather Squadron for all airports and meteorological stations in Kuwait and Iraq. We also obtained additional hourly visibility data from National Oceanic and Atmospheric Administration (NOAA) Integrated Surface Database (ISD, https://www.ncdc.noaa.gov/isd). Seventy-five percent of the visibility data in this study was from U.S. Air Force and 25% was from NOAA. We calculated average daily visibility from the available hourly data. The distribution of the 263 visibility monitoring stations available in Iraq and Kuwait during the study period is shown in Fig. S3.

#### 2.1.3. PM_2.5_ monitoring data

We obtained PM_2.5_ monitoring data in the study regions that were available over 3 different time periods and sites. A total of 1942 daily PM_2.5_ and concurrent visibility data were used in our model (Table S1). These included daily PM_2.5_ concentrations from U.S. Embassy Kuwait air quality monitor for 2017–2018 (https://kw.usembassy.gov/embassy/kuwait-city/air-quality-monitor/), PM_2.5_ concentrations we previously collected in three monitoring sites in Kuwait during 2004–2005 (Brown et al., 2008), and daily PM_2.5_ observations from two monitoring sites from 2017 to 2018 our group collected in Kuwait City.

#### 2.1.4. Meteorological data

Meteorological data were obtained from the fifth generation European Centre for Medium-Range Weather Forecasts reanalysis (ERA5) (https://cds.climate.copernicus.eu/cdsapp#!/search?type=dataset). ERA5 is a widely used global reanalysis, which has been verified by comparisons with observations (Hersbach et al., 2020). We considered 18 metrological variables, including temperature and dew point temperature at 2 m, u-wind (east–west component of the wind) speed at 10 m, v-wind (north–south component of the wind) speed at 10 m, instantaneous 10 m wind gust, total precipitation, surface pressure, downward UV radiation at the surface, cloud cover (high, medium, low, and total) evaporation, planetary boundary layer height, relative humidity at 1,000 hPa, vegetation cover (high and low), and forecast albedo (a measure of solar radiation)

#### 2.1.5. Dust and particle data

Since Iraq and Kuwait were largely covered by the desert and are impacted by intense and frequent dust storms, we assessed a number of surface dust related parameters as predictors of PM_2.5_ levels. Dust-related (e.g. “Dust surface Mass Concentration”, “Dust Extinction AOD”, “Dust Angstrom Parameter”) and additional particle parameters (e.g. “Organic Carbon Column Mass Density”, “Black Carbon Column Mass Density”) (Supplemental Table S2) were obtained from the Modern-Era Retrospective analysis for Research and Applications version 2 (MERRA-2) dataset (https://disc.gsfc.nasa.gov/datasets/M2TUNXAER_5.12.4/summary). MERRA-2 is an atmospheric reanalysis platform provided by NASA, which is agreed well with the results of other satellite observations and those of ground-based measurements (Ginoux et al., 2001; Rienecker et al., 2011; Colarco et al., 2010). MERRA-2 has a spatial resolution of 0.625°×0.5° (approximately 62.5 km × 50 km) and temporal resolution of one day.

#### 2.1.6. Land use data

Land use parameters including Normalized Difference Vegetation Index (NDVI), elevation, distance to the nearest industrial area, and traffic density were included as model predictors (Table S2). NDVI data were obtained from the NOAA Climate Data Record (CDR) of Normalized Difference Vegetation Index (NDVI), Version 4 (https://data.nodc.noaa.gov/cgi-bin/iso?id=gov.noaa.ncdc:C00813). This dataset was acquired by the Advanced Very High Resolution Radiometer (AVHRR) sensor at a spatial resolution of 5 km × 5 km and a temporal resolution of 1 day. Elevation of each grid cell was extracted from ETOPO1 Global Relief Model (https://www.ngdc.noaa.gov/mgg/global/global.html). ETOPO1 is a 1 arc-minute global relief model of Earth’s surface that integrates land topography and ocean bathymetry. Information regarding industrial areas was provided by the U.S. National Geospatial-Intelligence Agency. Since industrial emissions may influence the relationship between AOD and visibility, we calculated the distance from the center of each grid cell to the nearest industrial area for use as a potential adjustment variable. Road data were obtained through OpenStreetMap (https://www.openstreetmap.org) and traffic density in each 1 km2 grid cell was calculated by the total Highway road length in the grid cell.

### 2.2. Model

**Fig. 1.**
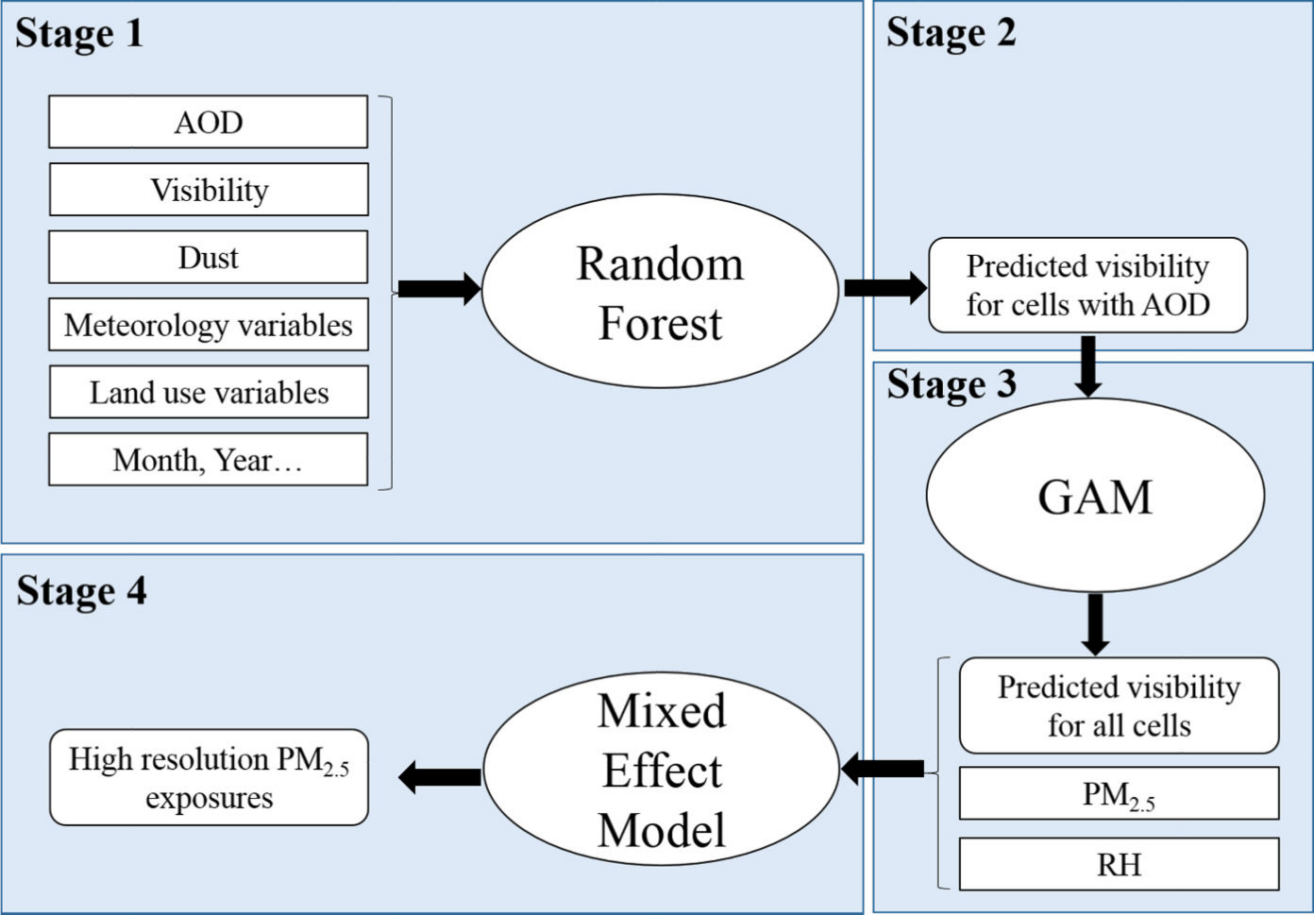
Flowchart of the four-stage modeling approach.

In order to estimate PM_2.5_ concentrations in each 1 km × 1 km grid cell, we developed a 4-stage model (Fig. 1).

#### 2.2.1. Stage 1

In stage 1, we used a random forest model to predict daily visibility based on MAIAC AOD, meteorological variables, MERRA-2 derived surface dust variables, and land use parameters. Random forest is a machine learning method for classification and regression which has been used to estimate PM_2.5_ (Huang et al., 2018; Di et al., 2019; Brokamp et al., 2017; Hu et al., 2017). The detailed predictors are shown in Supplemental Table S2. A total of 134,265 paired AOD-visibility observations were used for model training. We optimized model input parameters using a grid search approach (Di et al., 2019), with the input parameters listed in Supplemental Table S3. The final trained model was then used to predict daily visibility for each 1 km × 1 km grid cell with AOD.

We performed a 10-fold cross-validation by randomly dividing the visibility stations into 10%−90% splits (10 random groups). For each split, the model was trained with data from 90% of the visibility sites and was used to predict visibility in the 10% of sites not included in the training. This process was repeated 10 times, and the cross-validation R^2^ (CV R^2^) were computed.

#### 2.2.2. Stage 2

For grid cells with AOD but without visibility measurements, we used the Stage 1 model to estimate daily visibility for each grid.

#### 2.2.3. Stage 3

For grid cells missing both AOD and visibility measurements, a generalized additive mixed model (GAMM) was used to predict daily visibility based on the output from Stage 2 model (Kloog et al., 2014). This method was previously used in (Kloog et al., 2012). The model contains a smooth function of longitude and latitude, and a random intercept for each grid cell. A separate spatial surface was fit for each two-month period of each year to capture the temporal variations (Kloog et al., 2014). In addition, we included the mean daily visibility and a random cell-specific slope. The semi parametric regression model is shown as Eq. (1):

(1) $${\mathrm{PredVis}}_{ij}=\left(\alpha +\mu_{i}\right)+\left(\beta +v_{i}\right)MVis_{j}+\mathrm{Smooth}{\left(X,Y\right)}_{k(j)}+\epsilon_{i,j}\;{\left(u_{j}v_{j}\right)}^{\sim }\left((00),\Omega_{\beta }\right)$$

where *PredVis*_*ij*_ is the predicted visibility at grid-cell *i* on a day *j* from the stage 2 model; *MVis*_*j*_ is the mean value of predicted visibility from stage1 on a day *j* across the study region; *α* and *β* are the fixed intercept and fixed slope, respectively;*μ*_*i*_ and *v*_*i*_ are the random intercept and grid-cell specific random slope, respectively. X is longitude, and Y is latitude; “Smooth” is a thin plate spline function; and *k(j)* is the two-month period in which day *j* falls. To estimate the goodness of stage 3 model, we use the same cross-validation validation method as in stage 1.

#### 2.2.4. Stage 4

First, we fit a linear mixed effects model to describe the relationship between PM_2.5_ and visibility Because of the paucity of PM_2.5_ data in this region, we used data during 2017–2018 to train the model and used data during 2004–2005 for validation. Inverse of visibility and squared form of relative humidity were used as variables in the model based on the comparison of models using different combinations of variables (Text S1 and Table S5). The model can be expressed as:

(2) $$PM_{2.5i,j}=\left(\mu +\mu_{m}^{\prime }\right)+\left(\beta_{1}+\beta_{1m}^{\prime }\right)\left(\frac{1}{VIS_{i,j}}\right)+\beta_{2}RH_{i,j}+\beta_{3}RH_{i,j}^{2}\;\;+\varepsilon_{i,j}{\left(\mu_{m}^{\prime }\beta_{m}^{\prime }\right)}^{\sim }N((0,0),\Psi )$$

where PM_2.5i,j_ is the measurement daily PM_2.5_ concentrations for a PM_2.5_ site *i* on a day *j*; VIS is visibility; RH is relative humidity; *μ* is the fixed intercept; μ′_m_ is the month -specific random intercept, that is, the regression intercept in month *m*; β_1_ is the fixed slope for the inverse of visibility; β_1_′_m_ is the random slope for the inverse of visibility range in month j; β_2_ and β_3_ are the slopes for RH and squared form of relative humidity, respectively; ε_i,j_ is the error term at site i on day j. Ψ is the unstructured variance covariance matrix for the random effects.

Finally, we predicted PM_2.5_ concentration using Eq. (2) based on daily visibility for each 1 km × 1 km grid cell during 2001–2018 in Kuwait and Iraq. We used the results from the daily prediction model to calculate monthly and yearly PM_2.5_ averages.

All programming was implemented in R software version 3.5.0.

## 3. Results

**Fig. 2.**
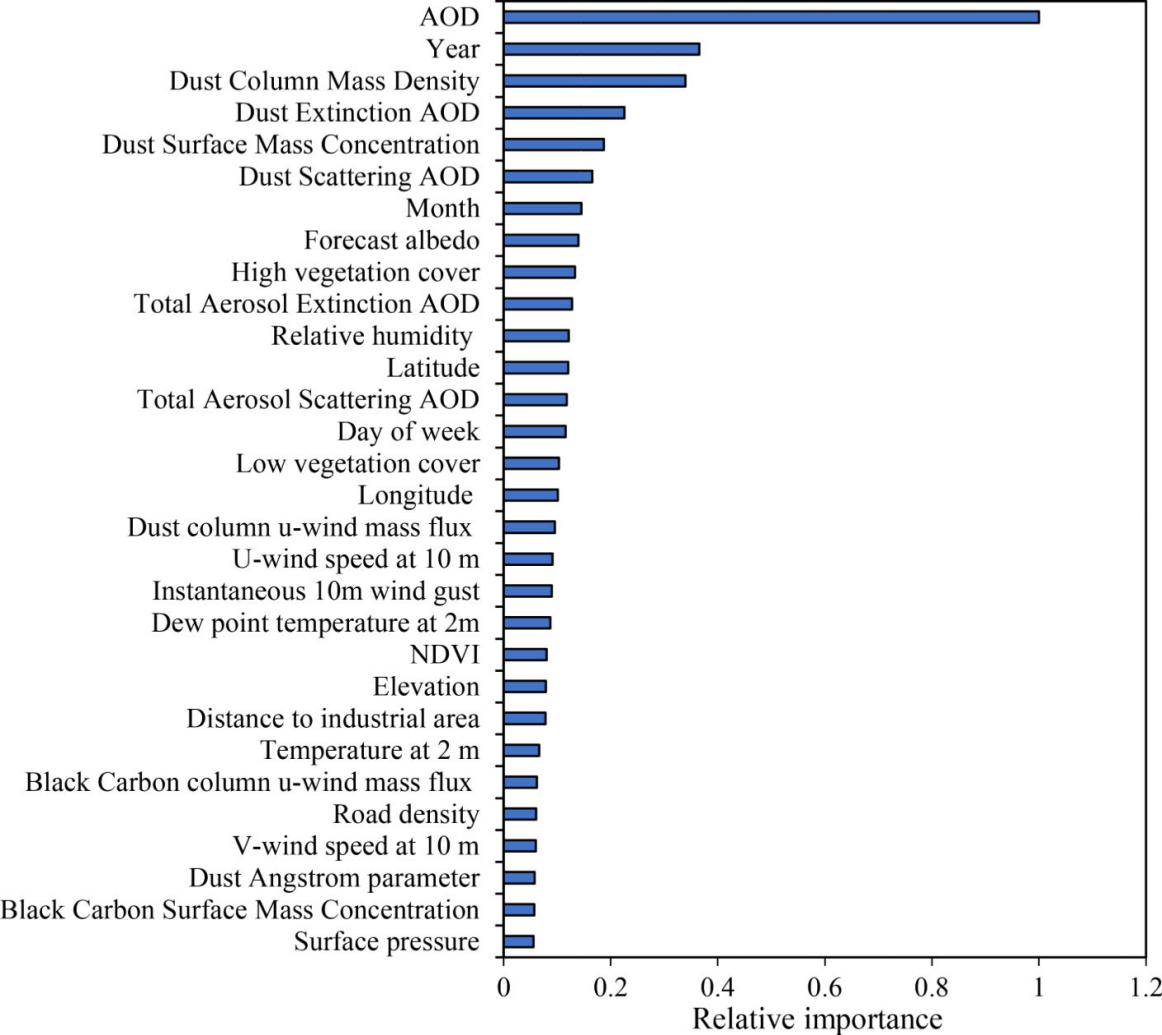
Top 30 most importance variables for the random forest model predicting daily visibility (x-axis: variable importance relative to the importance of AOD, calculated by function “h2o.varimp_plot” of R Package ‘h2o’).

The spatial distribution of yearly average MAIAC AOD downloaded from NASA for the study region is shown in Supplemental Fig. S4. The CV R^2^ between fitted and predicted daily visibility was 0.71 (Table S4). Fig. 2 illustrates the variable importance for the top 30 predictors in the stage 1 random forest model. MAIAC AOD, year, dust column mass density, dust extinction AOD, and dust surface mass concentration are the five most important predictors.

The stage 3 model performed well with a cross validation R^2^ of 0.68. A statistically significant association was found between visibility and daily PM_2.5_ in stage 4 model. This model resulted in a high CV R^2^ value of 0.70 in the years (2017–2018) and R^2^ value of 0.74 in 2004–2005, indicating good model fit (Table S5). The predicted and measured PM_2.5_ concentrations for stage 4 model are shown in Fig. S5.

**Fig. 3.**
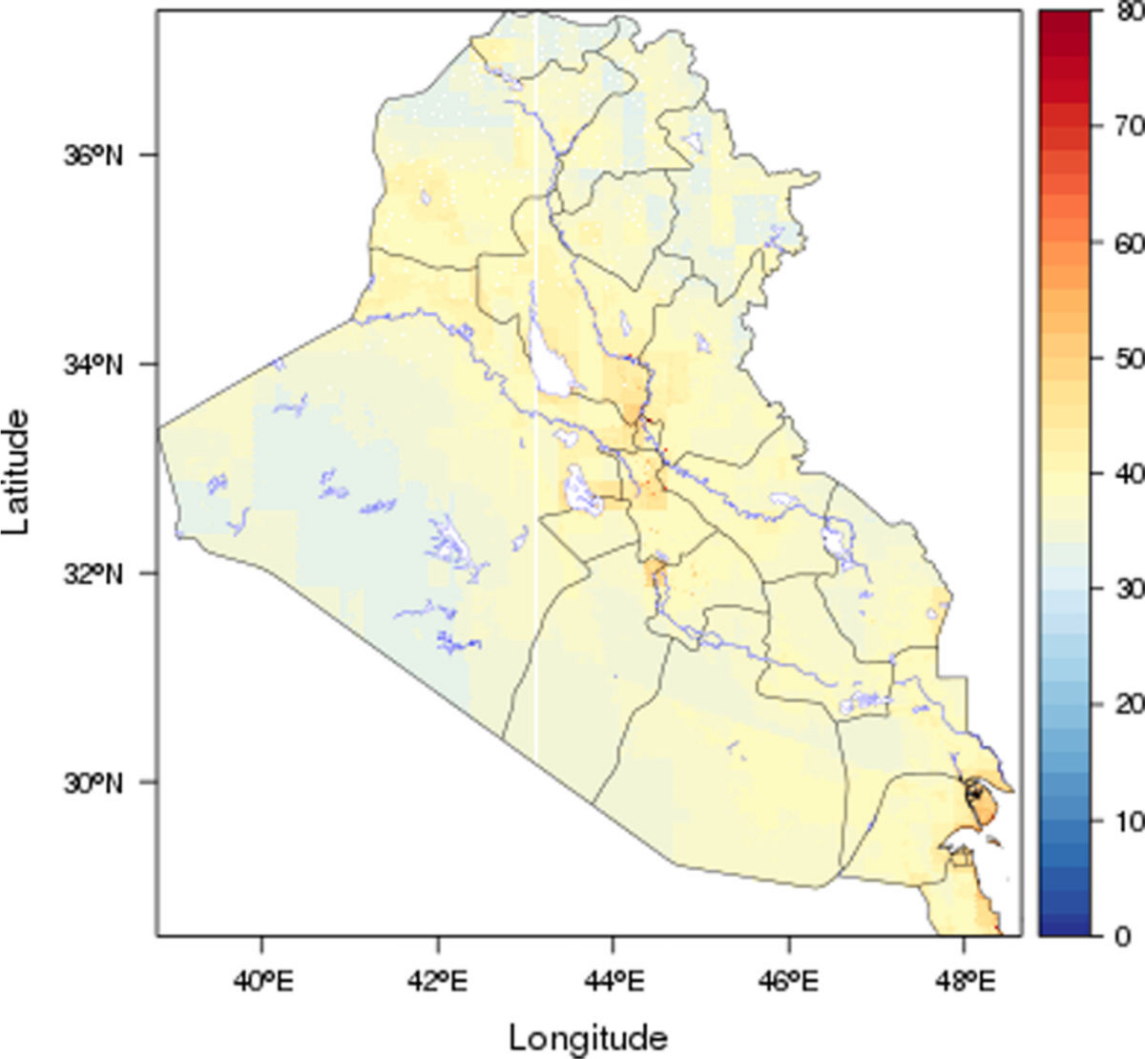
Mean PM_2.5_ concentrations (μg/m^3^) in each 1 km × 1 km grid during the entire modeling period predicted by our models (black line: boundary; blue line: water body).

**Fig. 5.**
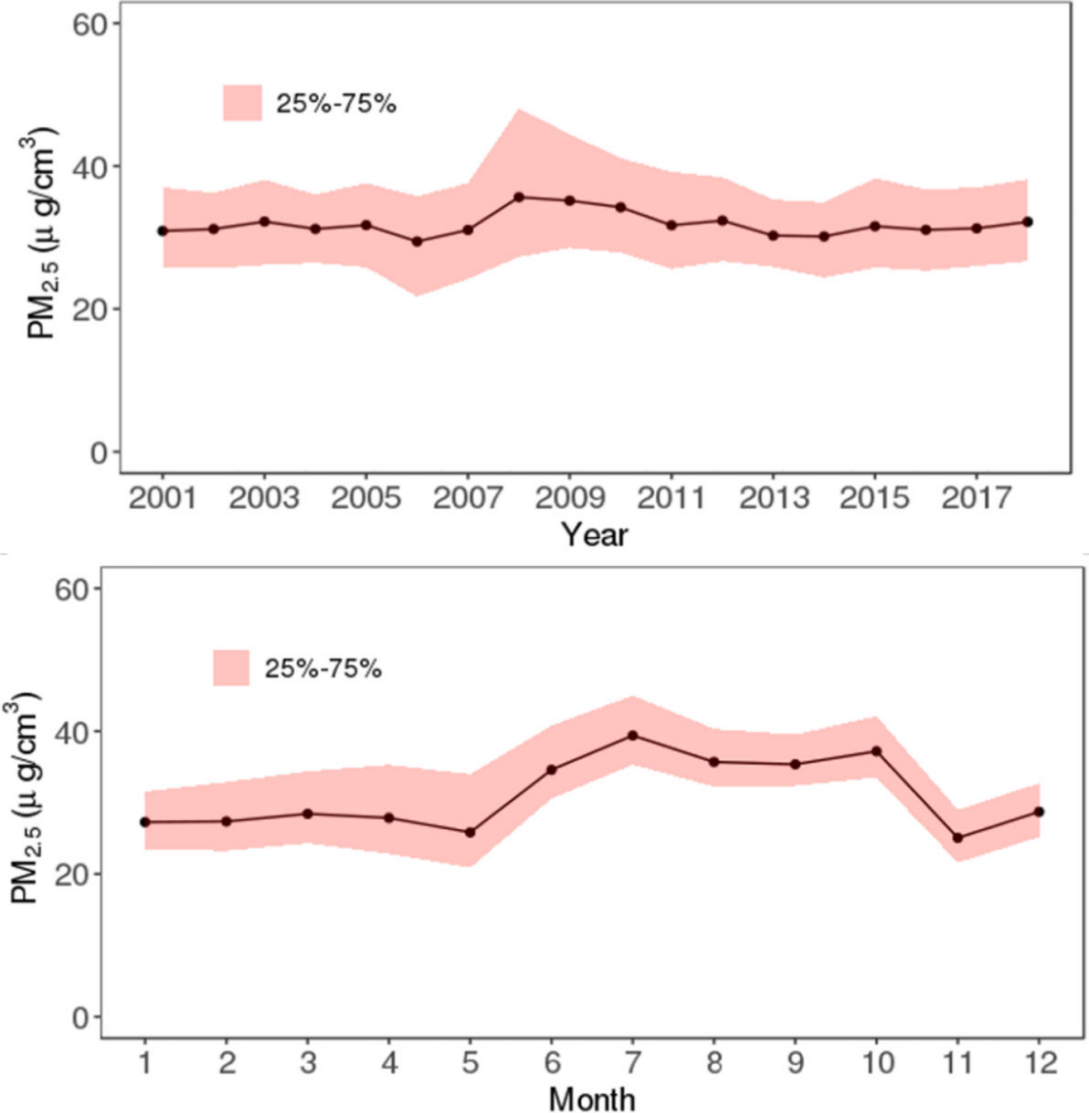
Annual and monthly average PM_2.5_ concentrations in Kuwait and Iraq.

We found considerable spatial and temporal variations over the study region. Fig. 3 shows the spatial distribution of the predicted PM_2.5_ concentrations in the study area, averaged over the full study period. The predictions for each year are shown in Fig. 4 and mean values for Iraq and Kuwait were 37 and 41 μg/m^3^, respectively. Annual and monthly median PM_2.5_ predictions for Iraq and Kuwait are shown in Fig. 5. Monthly average PM_2.5_ concentrations for each year are shown in Fig. S6.

In general, the PM_2.5_ concentrations in Kuwait were slightly higher than Iraq during 2001–2018. The annual PM_2.5_ concentration for Kuwait ranged from 36 to 49 μg/m^3^, and the values for Iraq ranged from 33 to 44 μg/m^3^. As expected, the predicted PM_2.5_ concentrations were higher in urban areas compared to rural areas. Some big cities such as Bagdad, Karbala, Najaf, and Diwaniya have annual average PM_2.5_ concentrations above 45 μg/m^3^. In contrast, Al-Anbar, the largest governorate west of Iraq (138,501 km^2^) which is mostly desert had low average PM_2.5_ concentrations, except for large urban areas in the east (Al-Fallujah and Al-Ramadi). PM_2.5_ levels in the study region also showed distinct temporal variability. The highest annual PM_2.5_ concentration for both Kuwait and Iraq were observed in 2008, followed by 2009. The lowest values for both Iraq and Kuwait were observed in 2014. It is possible that the extreme drought in 2008–2009 contributed to the high PM_2.5_ level in 2008 and heavy rain in the end of 2013 contributed to lower levels in 2014. By regions, the higher PM_2.5_ levels in 2008 and 2009 were predominantly in the urban areas. The monthly average predictions for Kuwait ranged from 32 to 49 μg/m^3^, and for Iraq ranged from 29 to 44 μg/m^3^. July had the highest predicted concentrations, and November had the lowest values. This is consistent with seasonal patterns of PM_2.5_ observations and indicates that PM_2.5_ pollution in the region has been a long-term problem.

As demonstrated by the considerable spatial and temporal variability over the region, deployers in Iraq and Kuwait experienced variability in PM_2.5_ exposures. Some bases were located in regions with lower PM_2.5_ concentrations such as in the Syrian Desert, which have average PM_2.5_ concentrations below 30 μg/m. However, many U.S. bases located in urban area and had very high PM_2.5_ concentrations. The weekly average PM_2.5_ at Baghdad International Airport, the US base in Balad, and Kuwait City International Airport are shown in Fig. S7. The weekly PM_2.5_ concentrations ranged to more than 150 μg/m^3^ for the bases in Baghdad and Balad in summer of 2008 and 2009.

## 4. Discussion

We developed a model to predict daily PM_2.5_ exposures at 1 km × 1 km resolution for Iraq and Kuwait, where the monitoring system is very limited. Our results indicated that this region had very high PM_2.5_ levels, which varied by location and year.

The annual mean predicted PM_2.5_ concentrations for Kuwait (41 μg/m^3^) and Iraq (37 μg/m^3^) from 2001 to 2018 were considerably higher than the U.S. National Air Quality Standard of 12 μg/m^3^ and the WHO guideline of 10 μg/m^3^. Very few studies conducted PM_2.5_ measurements in Kuwait or Iraq. Engelbrecht et al. (2009) collected PM_2.5_ samples approximately four times per month at five sampling sites in Iraq and four sampling sites in Kuwait in 2006. Al-Hemoud et al. (2018a) assessed daily air concentration of PM_10_ for the year 2012 in a suburban area of Kuwait. Al-Hemoud et al. (2018b) and Al-Hemoud et al. (2019) collected PM_2.5_ measurements in three monitoring stations in Kuwait from 2014 to 2017. Our estimates have the same spatial distribution with the PM_2.5_ samples of Engelbrecht et al. (2009). Our predictions and the PM_10_ in Al-Hemoud et al. (2018a) have the same seasonal pattern. The PM_2.5_ concentrations predicted in this study are consistent with a previous WHO air pollution database (WHO, 2018). However, the PM_2.5_ concentrations in (Al-Hemoud et al., 2019) (34–80 μg/m^3^) are generally higher than our results, possibly because they selected hourly PM_2.5_ values above a minimum background concentration of 8.83 μg/m^3^ to report.

Our study has some major findings that are of public health significance. In previous studies, the AOD-PM_2.5_ relationship was directly used to examine the spatial distribution of PM_2.5_. However, for many countries, there are no available historical PM_2.5_ measurements. Our novel exposure assessment approach indicates it possible to assess air pollution exposures in countries without extensive PM_2.5_ monitoring locations, and without extensive PM_2.5_ historical data. And the other predictors used in our model are predominantly from global public datasets, thus our method could be applied in other countries. Secondly, the random forest model approach provided the relative importance of each factor associated with historical PM_2.5_ concentrations to assess exposures in this region or similar areas. For example, in addition to AOD, we found that surface dust related variables are also associated with PM_2.5_ in arid environments (Li et al., 2020). The seasonal and monthly trends of PM_2.5_ are consistent with the seasonal occurrence of dust storms in the region, demonstrating the impact of dust storms on the PM_2.5_ model predictions. (Al-Hemoud et al., 2017; Al-Hemoud et al., 2018a; Li et al., 2020).

A limitation of our approach is that the stage 4 model regression results was based on PM_2.5_ measurement data collected at three sampling sites for two years because of the paucity of PM_2.5_ data in this region. We used the PM_2.5_ data collected 2017–2018 to train the model and used data collected 2004–2005 for validation, which could have potentially influenced the accuracy of historical predictions. However, the relationship between PM_2.5_ and visibility is relatively stable under specific conditions of relative humidity as in Iraq and Kuwait (Zhao et al., 2017; Liu et al., 2017). It has also been shown that PM_2.5_ particles in Iraq and Kuwait exhibit similar hygroscopic properties (Abdeen et al., 2014). In support of our approach is that the cross validation of the 2004–2005 PM_2.5_ data indicated good model fit. Another limitation is that the AOD data used in this study has nonrandom missing data (such as days with heavy cloud clover) that was accounted for by modeling, and the local crossing time of the AOD data were relatively fixed between 10:30 am and 1:30 pm. Although these factors could result in systematic bias of the PM_2.5_ predictions, the model would still be internally consistent.

To the best of our knowledge, this is the first model to estimate historical PM_2.5_ concentrations at a high resolution in Iraq and Kuwait that takes advantage of all available measurement data. This high-resolution model makes it possible to assess the health effects of PM_2.5_ exposures both in the native populations and the military personnel. This study is also helpful to the construction of regional environmental policies in areas with different pollution levels.

## Supplementary Material

supplement

## Figures and Tables

**Fig. 4a.**
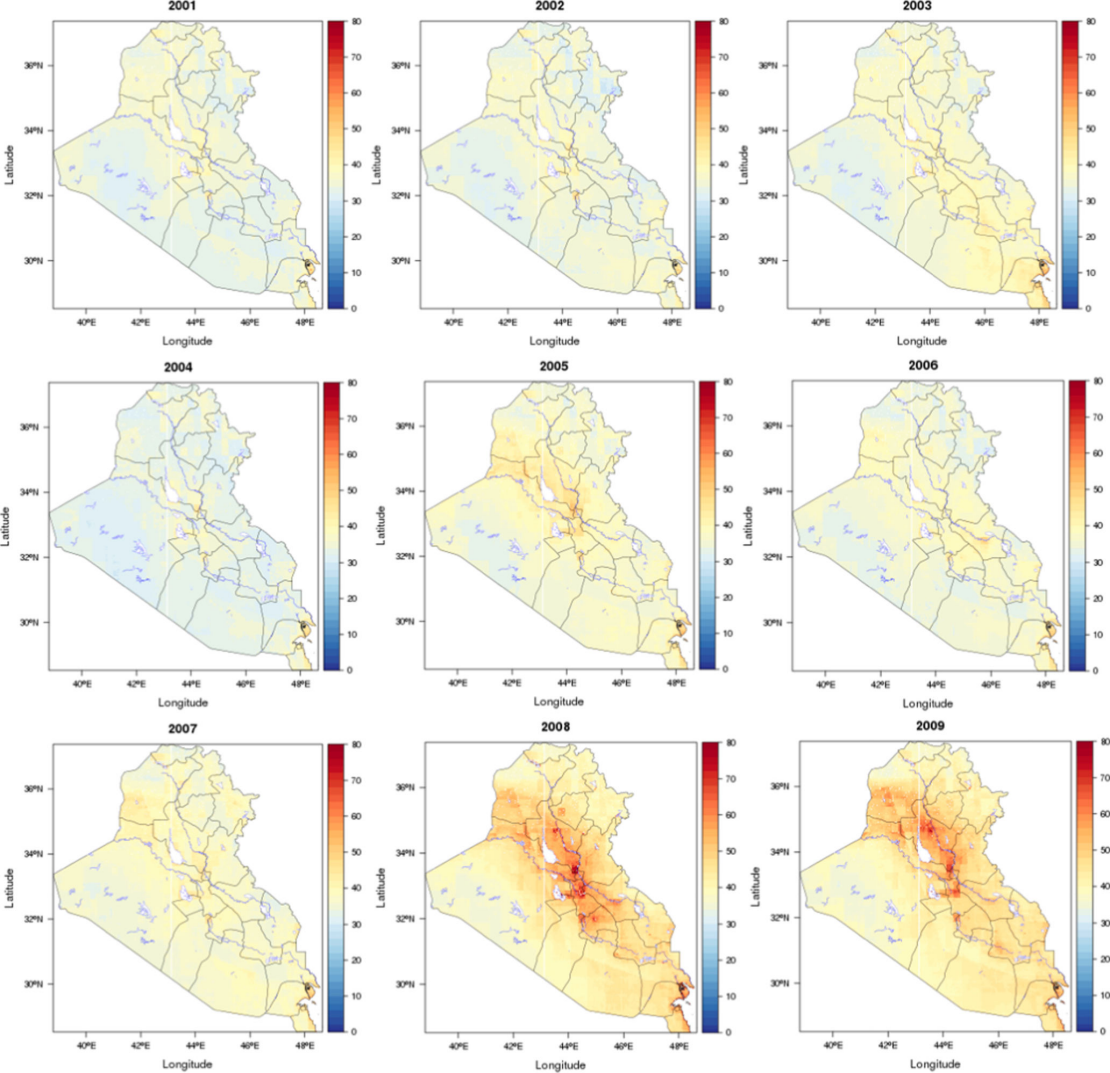
Spatial distribution of predicted mean PM_2.5_ concentrations (μg/m^3^) for each year (2001–2009; Black line: boundary; Blue line: water body).

**Fig. 4b.**
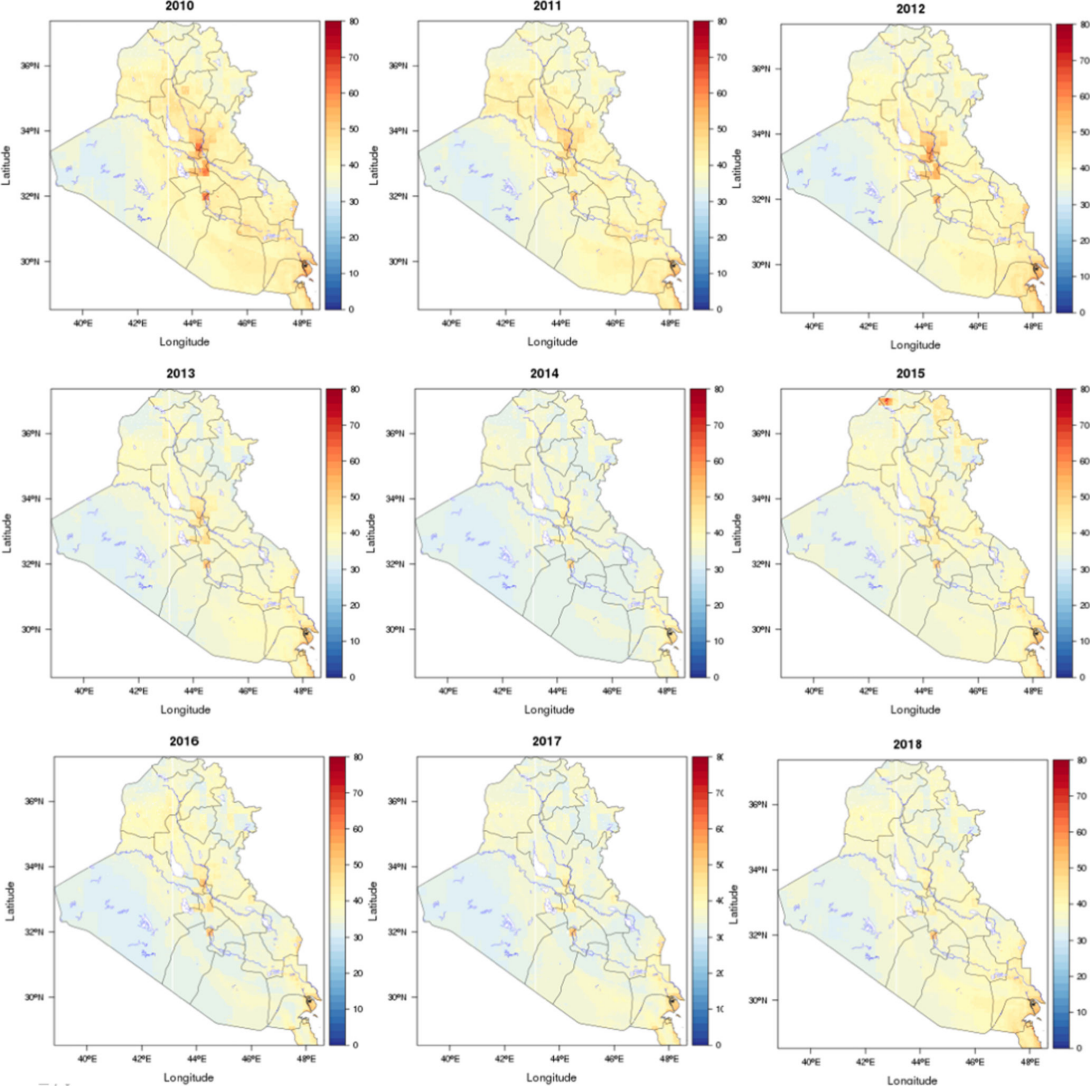
Spatial distribution of predicted mean PM_2.5_ concentrations (μg/m^3^) for each year (2010–2018; Black line: boundary; Blue line: water body).
