# Supplementary material for: Estimation of ambient PM2.5 in Iraq and Kuwait from 2001 to 2018 using machine learning and remote sensing
Source: Environ Int. Author manuscript; Available in PMC 2022 Jun 1. (PMC8023768; doi:10.1016/j.envint.2021.106445)
Supplement: supplement [file NIHMS1678488-supplement-supplement.docx]

# Supplementary Information

# *for*

**Estimation of ambient PM2.5 in Iraq and Kuwait from 2001 to 2018 using machine learning and remote sensing**

Jing Li,^†^ Eric Garshick,^‡,^^§^ Jaime E. Hart,^†,§^ Longxiang Li,^†^ Liuhua Shi ^†,∥^, Ali Al-Hemoud,^#^ Shaodan Huang,^† ,*^ and Petros Koutrakis^†^

^†^ Department of Environmental Health, [Harvard T.H. Chan School of Public Health](https://www.google.com/url?sa=t&rct=j&q=&esrc=s&source=web&cd=1&cad=rja&uact=8&ved=0ahUKEwiY2bKvzZLSAhUG6GMKHZqACEEQFggaMAA&url=https%3A%2F%2Fwww.hsph.harvard.edu%2Fpetros-koutrakis%2F&usg=AFQjCNGG7yPRcpWLLFuQxcrbZ2RZc0YuWA&sig2=A2Ks24lul6YnLozL_T2TYg), Boston 02115, USA

^‡^ Pulmonary, Allergy, Sleep, and Critical Care Medicine Section, Medical Service, VA Boston Healthcare System, Boston, MA 02132, USA

^§^ Channing Division of Network Medicine, Department of Medicine, Brigham and Women’s Hospital and Harvard Medical School, Boston, MA, 02115, USA

^∥^ Department of Environmental Health, Rollins School of Public Health, Emory University, Atlanta, Georgia, 30322, USA

^#^ Crisis Decision Support Program, Environment and Life Sciences Research Center, Kuwait Institute for Scientific Research, Safat 13109, Kuwait

^*^ Corresponding Author Telephone: +1 (617) 480-6288; Fax: +1 (617) 230-5242

E-mail: shhuang@hsph.harvard.edu.

**Text S1 Stage 4 model**

The inclusion of inverse of visibility (1/VIS) and the squared form of relative humidity (RH^2^) as variables describing the association between visibility and PM_2.5_ is based on the comparison of models using different combinations of variables(Liu et al., 2017). We evaluated the performance of models using different combinations of variables. Validation results for different models are shown in Table S5. The relationship between visibility and PM_2.5_ is often parameterized as either an inverse or on a logarithmic scale(Wang et al., 2019). We tested both inverse and natural log of visibility, and the model with the inverse of visibility had the best performance in both cross validation and validation of historical estimates. We also assessed the square of relative humidity since the scattering efficiency of PM_2.5_ has been shown to increase quadratically with increasing relative humidity (Malm and Day, 2001;Masri et al., 2017). After including the square of relative humidity, the cross validation and historical estimates for visibility improved. Incorporating other meteorological data such as wind speed, temperature and relative humidity did not improve the model performance (Table S5).

Table S1. PM_2.5_ samples in Kuwait used to calibrate modeled visibility to PM_2.5._

| **Sampling site** | **Longitude, Latitude** | **Sampling date** | **Number of Samples** |
| --- | --- | --- | --- |
| Central site | 29.33,47.97 | 2004-2005 | 439 |
|  |  | 2017-2019 | 395 |
| Northern site | 29.77,47.77 | 2004-2005 | 40 |
| Southern site | 28.96,48.16 | 2004-2005 | 62 |
|  |  | 2017-2019 | 350 |
| U.S. Embassy Kuwait | 29.31,48.04 | 2017-2018 | 656 |

Table S2. Predictors used in Stage 1 model.

| Predictor | Data source |
| --- | --- |
| AOD | MAIAC |
| NDVI | AVHRR |
| Temperature at 2 m | ERA5 |
| U-wind speed at 10 m | ERA5 |
| V-wind speed at 10 m | ERA5 |
| Instantaneous 10m wind gust | ERA5 |
| Dew point temperature at 2m | ERA5 |
| Total precipitation | ERA5 |
| Surface pressure | ERA5 |
| Downward UV radiation | ERA5 |
| Evaporation | ERA5 |
| Elevation | ETOPO1 |
| Year | -- |
| Month | -- |
| Day of week | -- |
| Total cloud cover | ERA5 |
| Low cloud cover | ERA5 |
| Medium cloud cover | ERA5 |
| High cloud cover | ERA5 |
| High vegetation cover | ERA5 |
| Low vegetation cover | ERA5 |
| Forecast albedo | ERA5 |
| Planetary boundary layer height | ERA5 |
| Land cover type | ERA5 |
| Relative humidity | ERA5 |
| Longitude | -- |
| Latitude | -- |
| Distance to industrial area | U.S. National Geospatial-Intelligence Agency |
| Road density | OpenStreetMap |
| Organic Carbon Column Mass Density | MERRA2 |
| Dust Surface Mass Concentration | MERRA2 |
| Dust Extinction AOD | MERRA2 |
| Dust Scattering AOD | MERRA2 |
| Dust Column Mass Density | MERRA2 |
| Dust column u-wind mass flux | MERRA2 |
| Dust column v-wind mass flux | MERRA2 |
| Dust Angstrom parameter | MERRA2 |
| Total Aerosol Extinction AOD | MERRA2 |
| Total Aerosol Scattering AOD | MERRA2 |
| Black Carbon column u-wind mass flux | MERRA2 |
| Black Carbon column v-wind mass flux | MERRA2 |
| Black Carbon Angstrom parameter | MERRA2 |
| Black Carbon Scattering AOT | MERRA2 |
| Black Carbon Extinction AOT | MERRA2 |
| Black Carbon Column Mass Density | MERRA2 |

Table S3. Input parameters* for the Stage 1 model.

| **Parameter Name** | **Final Value after Training** |
| --- | --- |
| Number of trees | 1000 |
| Sample rate | 0.9 |
| Maximum tree depth | 40 |
| Number of bins for numerical columns | 1024 |
| Number of bins for categorical columns | 1024 |

* See R Package ‘h2o’for a description of these input parameters

Table S4. Cross-validated R^2^ for stage 1 predictions.

| **Simulation** | **CV R^2^** |
| --- | --- |
| 1 | 0.71 |
| 2 | 0.85 |
| 3 | 0.71 |
| 4 | 0.77 |
| 5 | 0.65 |
| 6 | 0.70 |
| 7 | 0.68 |
| 8 | 0.60 |
| 9 | 0.68 |
| 10 | 0.80 |
| Mean | 0.71 |

Table S5. Validation of models for stage 4 using different combinations of variables.

| Model variables | Overall Cross validation R^2^ in the modeling year (2017-2018) | Validation R^2^ of historical estimates at daily levels (2004-2005) |
| --- | --- | --- |
| VIS | 0.600 | 0.635 |
| 1/VIS | 0.664 | 0.723 |
| lnVIS | 0.656 | 0.698 |
| RH+1/VIS | 0.662 | 0.724 |
| WS+1/VIS | 0.667 | 0.724 |
| TEMP+1/VIS | 0.630 | 0.685 |
| RH+TEMP+1/VIS | 0.669 | 0.722 |
| RH+WS+1/VIS | 0.637 | 0.705 |
| TEMP+WS+1/VIS | 0.673 | 0.721 |
| RH+TEMP+WS+1/VIS | 0.632 | 0.686 |
| RH+RH^2^+TEMP+WS+1/VIS | 0.706 | 0.743 |
| RH+RH^2^+1/VIS | 0.698 | 0.745 |


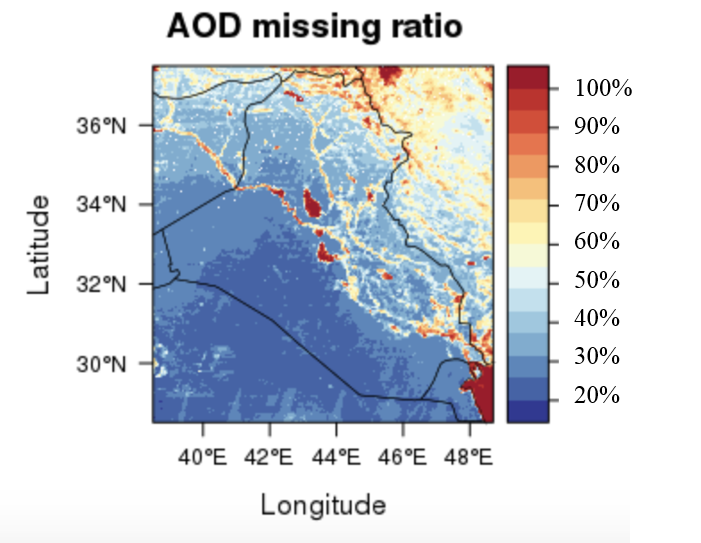


Figure S1. The percentage of the missing AOD values in each grid cell from 2001-2018. The darker brown values indicating a very high percentages of missing data are over bodies of water that do not contribute to the prediction model.

Figure S2. The percentage of the missing AOD values by month and by year.


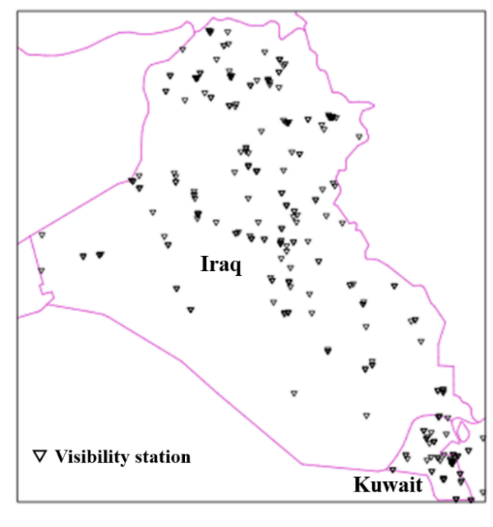


Figure S3. Map of the study area (Iraq and Kuwait) with visibility stations (Pink line: boundary; black triangle: visibility station).


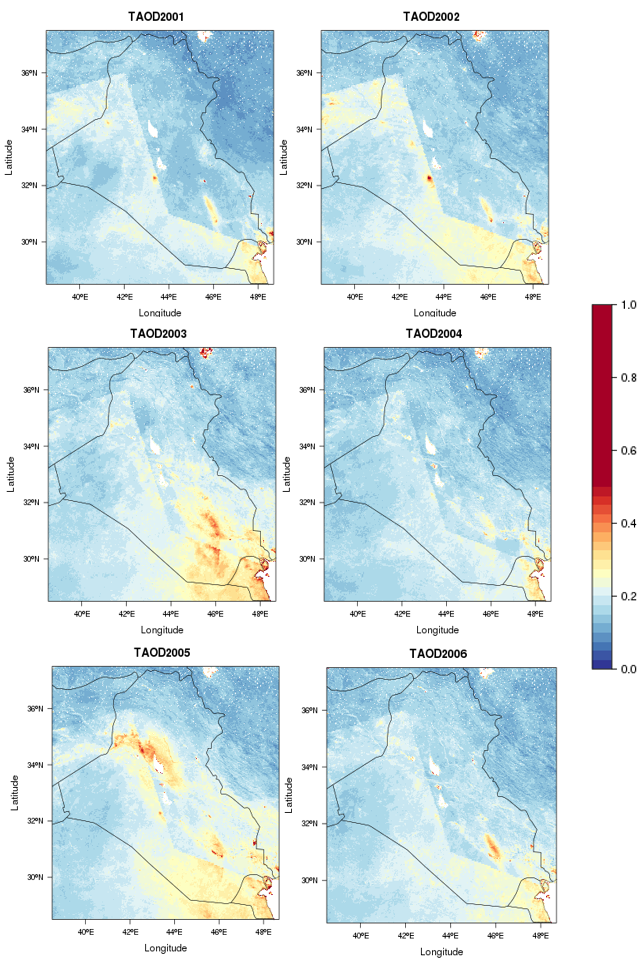


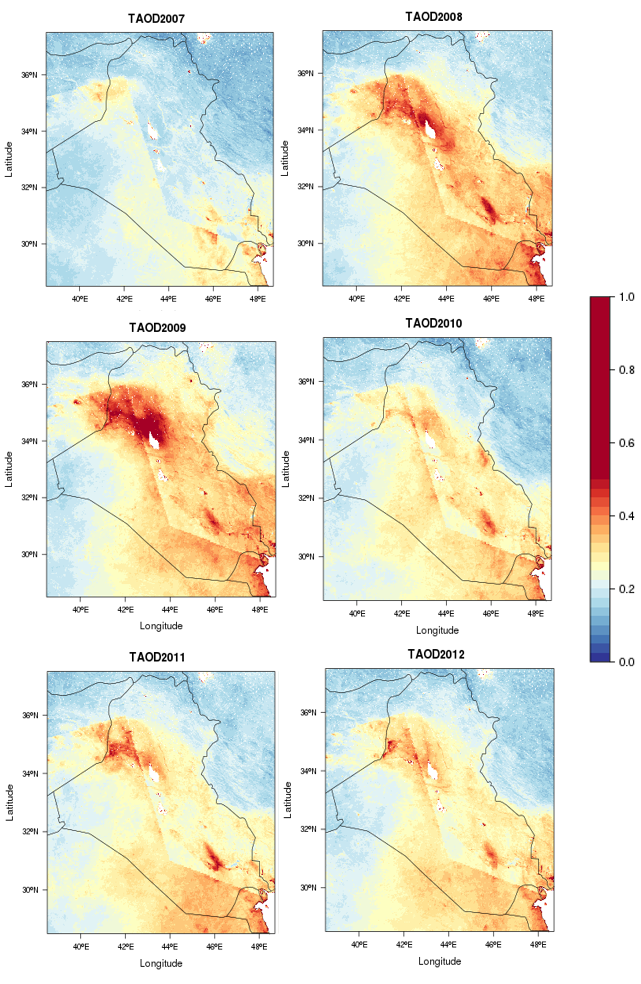


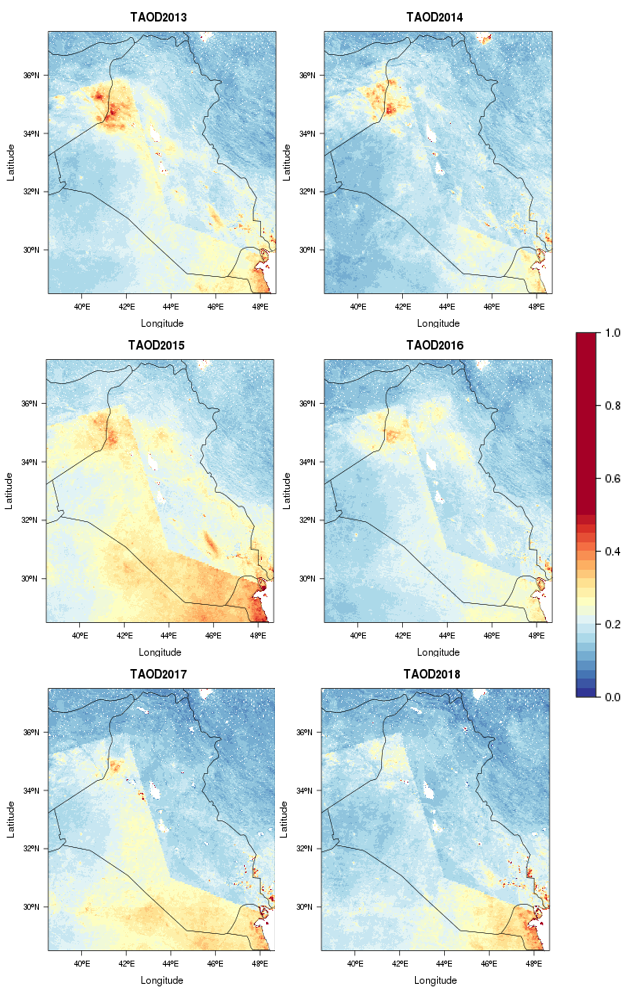


Figure S4. Spatial distribution of yearly average MAIAC terra AOD for the study region.


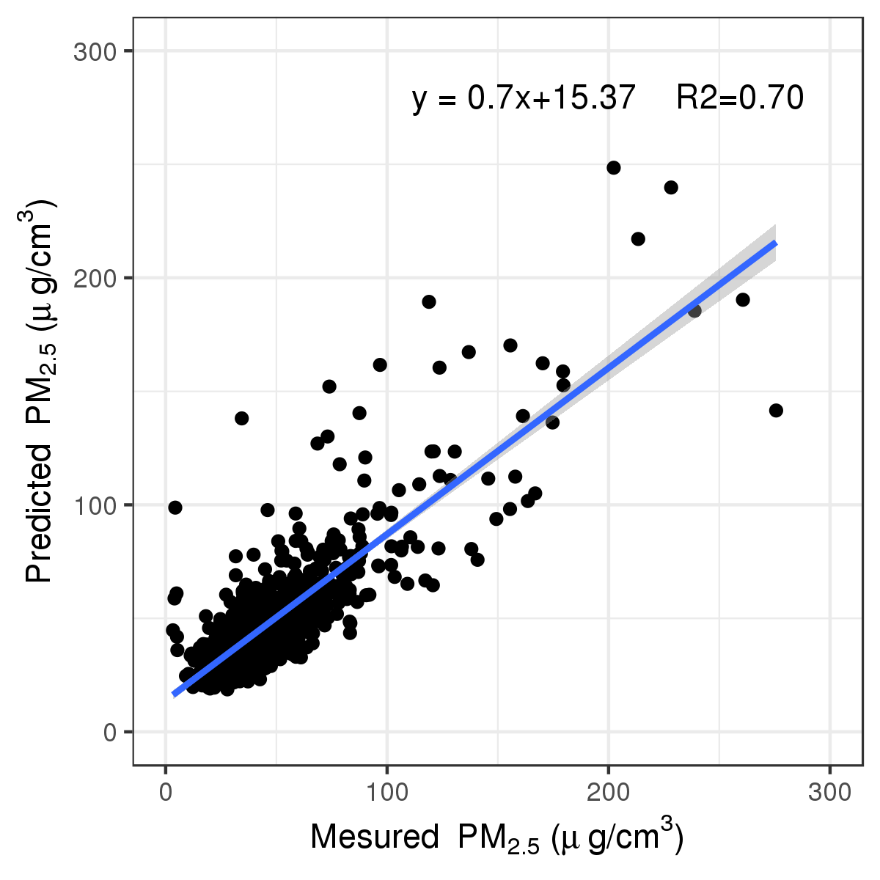


Figure S5. Relationship between predicted and measured PM_2.5_ concentrations of stage 4 model.


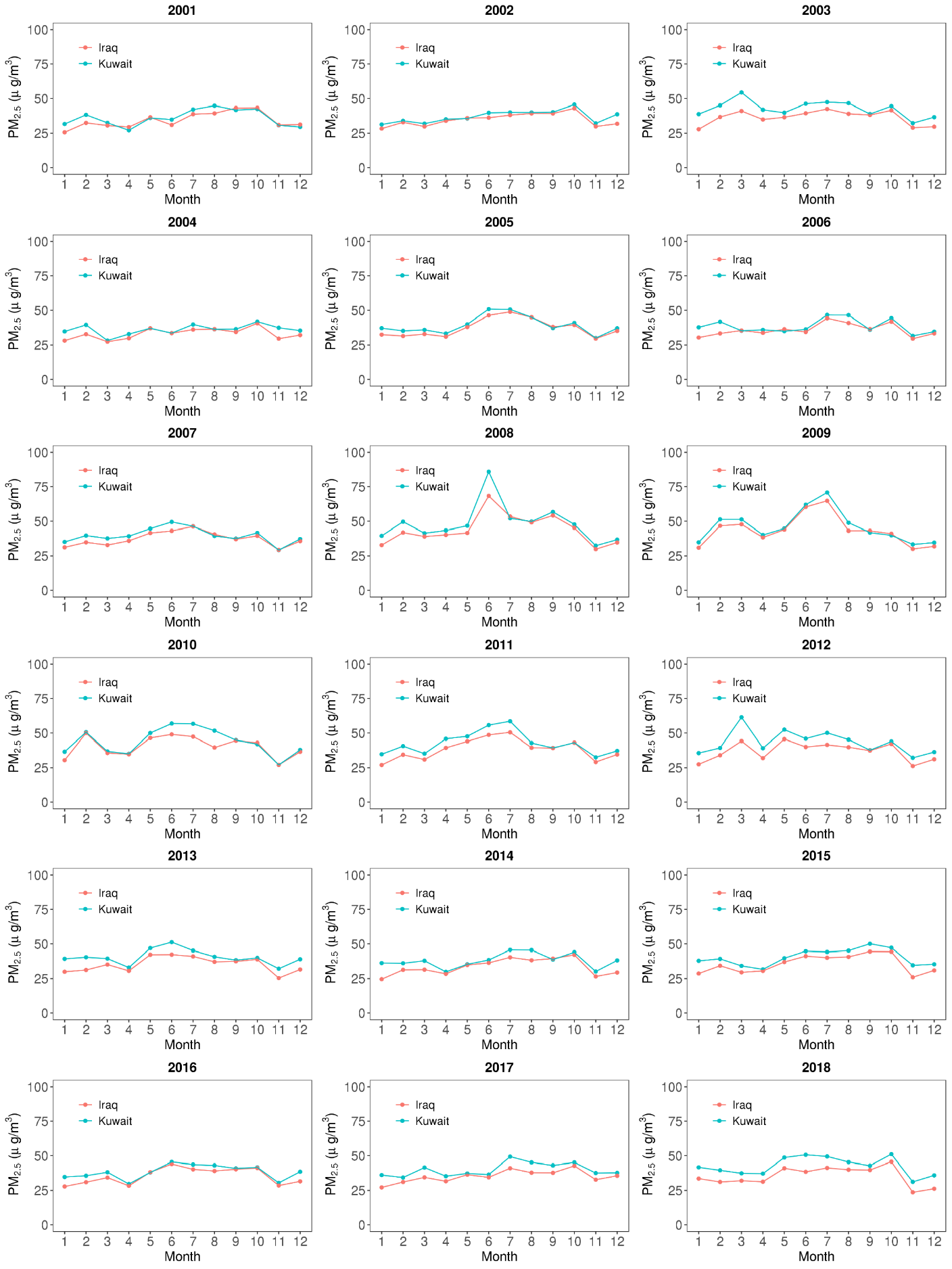


Figure S6. Monthly average PM_2.5_ concentrations for each year by country.


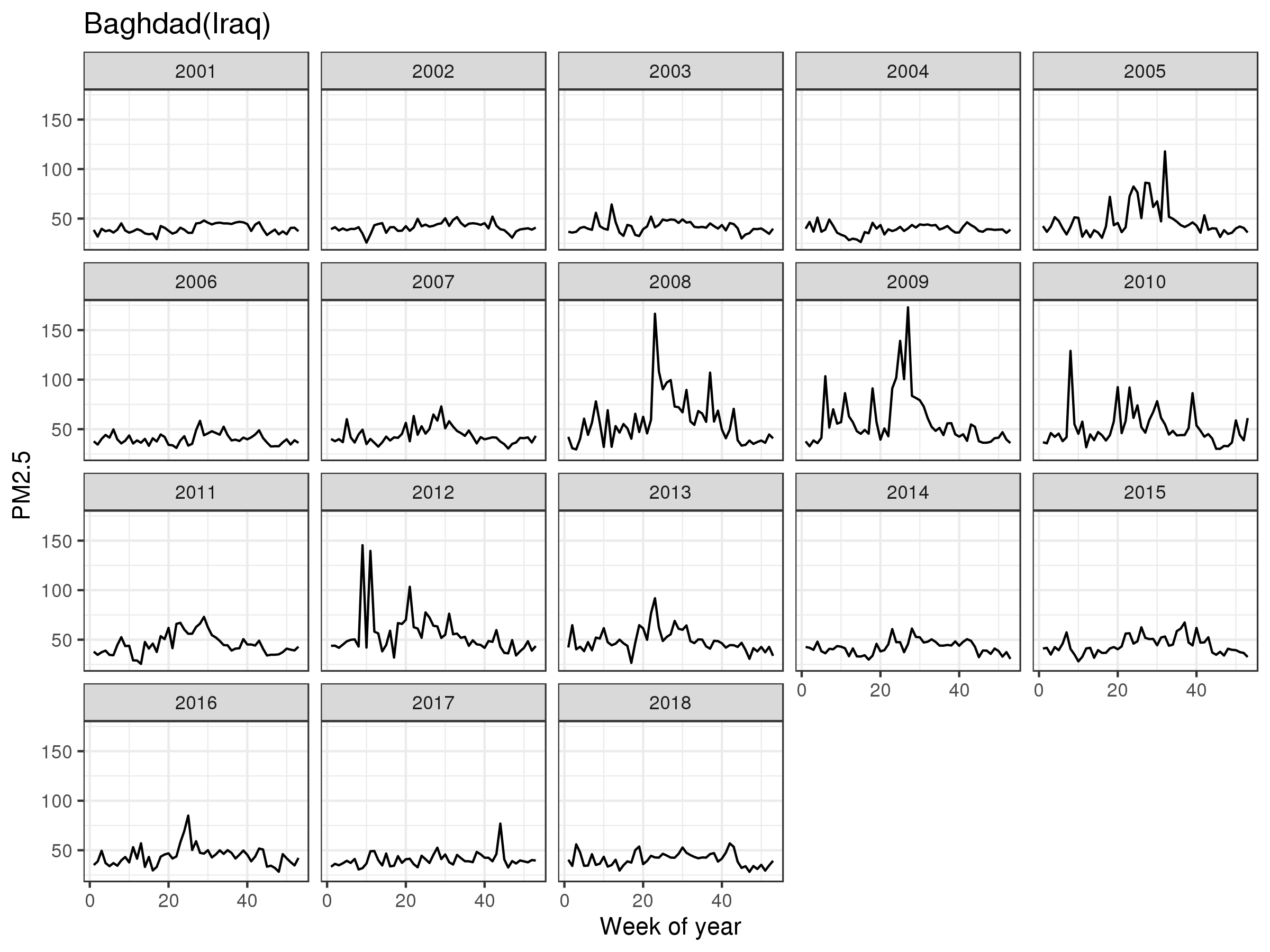


Figure S7a. Weekly average PM_2.5_ concentrations at Baghdad International Airport.


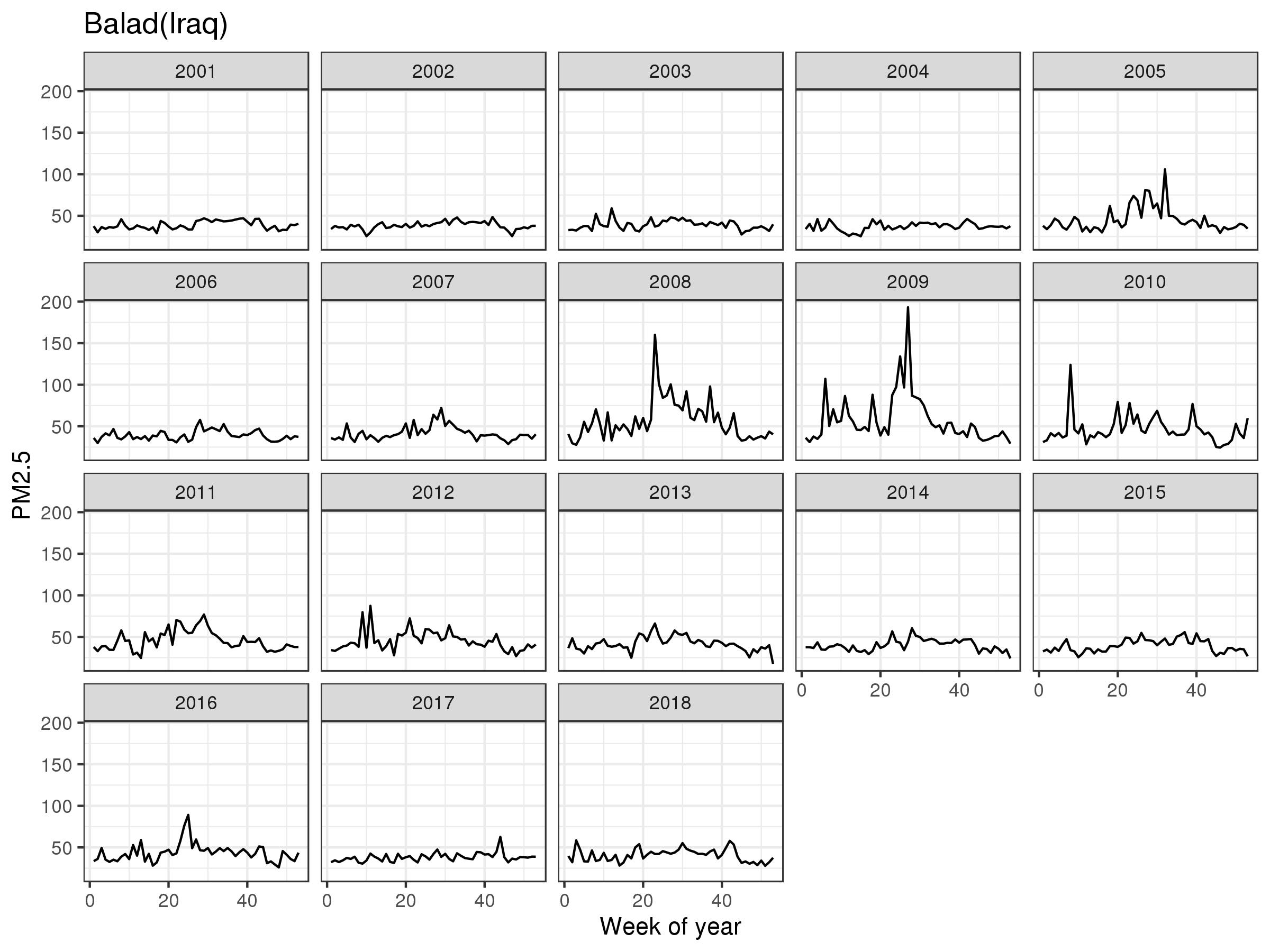


Figure S7b. Weekly average PM_2.5_ concentrations for the US base at Balad.


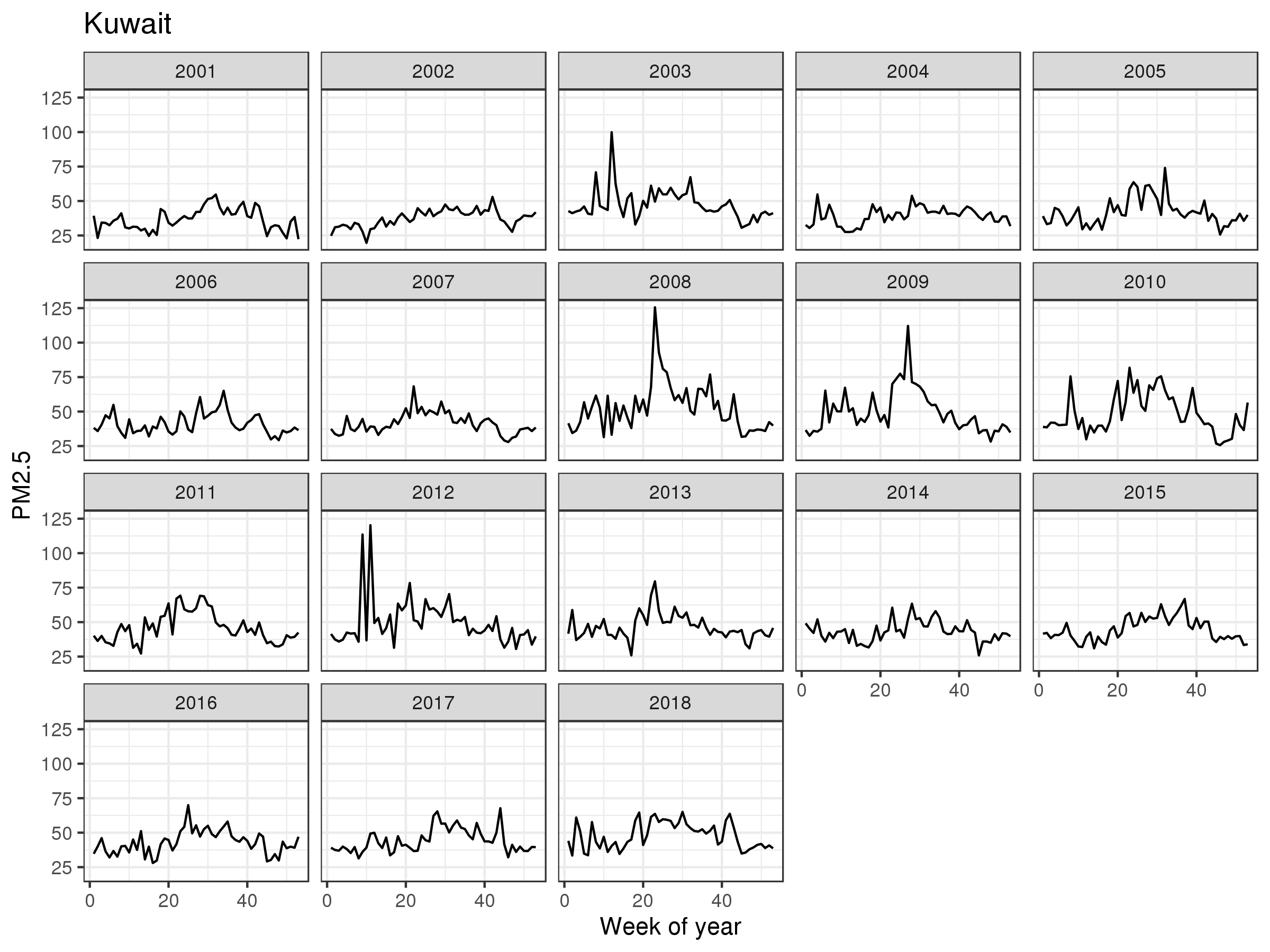


Figure s7c. Weekly average PM_2.5_ concentrations at Kuwait International Airport

References:

Liu, M., Bi, J., and Ma, Z.: Visibility-Based PM2.5 Concentrations in China: 1957-1964 and 1973-2014, Environ. Sci. Technol., 51, 13161-13169, 10.1021/acs.est.7b03468, 2017.

Malm, W. C., and Day, D. E.: Estimates of aerosol species scattering characteristics as a function of relative humidity, Atmos. Environ., 35, 2845-2860, 10.1016/s1352-2310(01)00077-2, 2001.

Masri, S., Garshick, E., Hart, J., Bouhamra, W., and Koutrakis, P.: Use of visual range measurements to predict fine particulate matter exposures in Southwest Asia and Afghanistan, J

Air Waste Manag Assoc, 67, 75-85, 10.1080/10962247.2016.1243169, 2017.

Wang, X., Zhang, R., and Yu, W.: The Effects of PM2.5 Concentrations and Relative Humidity on Atmospheric Visibility in Beijing, J. Geophys. Res.-Atmos., 124, 2235-2259, 10.1029/2018jd029269, 2019.
